# Effects of early and later life environmental enrichment and personality on attention bias in pigs (*Sus scrofa domesticus*)

**DOI:** 10.1007/s10071-019-01287-w

**Published:** 2019-06-27

**Authors:** Lu Luo, Inonge Reimert, Elske N. de Haas, Bas Kemp, J. Elizabeth Bolhuis

**Affiliations:** 1 Adaptation Physiology Group, Department of Animal Sciences, Wageningen University and Research, Wageningen, The Netherlands; 2 Present Address: Department of Animals in Science and Society, Utrecht University, 3584 CL Utrecht, The Netherlands

**Keywords:** Pigs, Environmental enrichment, Attention bias, Affective states, Coping style, Early life

## Abstract

We investigated effects of early and later life housing on attention bias, as an indicator of affective state, in pigs differing in coping style [reactive (LR) vs. proactive (HR)]. Pigs (*n* = 128) in barren or enriched housing from birth (B1 vs. E1) that experienced either a switch in housing at 7 weeks of age or not (creating B1B2, B1E2, E1E2, and E1B2 treatments), were studied in a 180-s attention bias test at 11 weeks. Pigs exposed to a 10-s-auditory-and-sudden-motion threat in the test arena paid more attention to the location of the threat, were more vigilant, showed less eating, more walking and were more likely to utter high-pitched vocalisations than non-threat pigs. During threat presence, HR pigs from post-switch enriched housing (E2-HR, i.e., B1E2 + E1E2) showed more vigilance but less exploration than others. After threat removal, no effects were found on time spent paying attention to the threat, vigilance, and eating, but E2-HR pigs paid attention to the threat more frequently, were more likely to utter high-pitched vocalisations and walked more compared to (part of) other groups, suggesting the most negative affective state in these animals. E2 pigs grunted more than B2 pigs. Thus, current housing, but not early life housing, affected behaviour in a personality-dependent manner in this attention bias test. Housing effects were opposite to expectation, possibly due to the short-term effect of the relative contrast between the home pens of the pigs and the test room. This potentially overruled putative long-term effects of environmental conditions on attention bias.

## Introduction

Affective states can influence cognitive processes, such as attention, memory, decision-making, and judgement. For example, a negative affective state can change expectations for the future and make animals more pessimistic (Paul et al. 2005), alter the motivation to consume a reward, enhance sensitivity to reward loss (Chaby et al. 2013), and increase attention towards negative stimuli (Lee et al. 2016). The form of cognitive bias most widely studied in non-human animals is judgement bias, in which the affective state of the animal influences its interpretation of ambiguous situations or cues (Mendl et al. 2009). In judgement bias tasks, animals need to be trained to discriminate between and respond to a positive and a negative stimulus before ambiguous stimuli are presented (Harding et al. 2004). This can be time-consuming and often not all animals can be successfully trained for the task (e.g., Verbeek et al. 2014). Besides, the training itself could be seen as cognitive enrichment and, thereby, may influence affective state and overrule the mood-influencing factors under study (e.g., Roelofs et al. 2016). Attention bias tests, which do not require training, have been suggested to offer a faster and more practical method to assess negative affective states, like anxiety, in animals (Lee et al. 2016).

Negative affective states, such as anxiety, can result in an attention bias towards a potential threat (Lee et al. 2018). Indeed, in humans, affect-driven attention bias has been demonstrated, as individuals in high states of anxiety show greater attention towards threatening stimuli than non-anxious individuals (Bradley et al. 1995, 1997). Also depression (Monk et al. 2018b) and chronic stress (Sipos et al. 2014) have been linked to altered threat perception. Attention bias tests have been conducted in a range of animals, as well. For instance, sheep in an anxious state induced by an anxiogenic drug responded with increased vigilance and paid more attention to the previous location of a short-lived threat (a predator) than sheep in a reduced anxious state induced by an anxiolytic substance (Lee et al. 2016). They also showed a reduced willingness to consume the feed that was offered in the test, even after the threat had disappeared. Similarly, starlings that had been deprived of water bathing (and, therefore, likely in a negative affective state), showed more vigilance and less feeding following playback of an alarm call (Brilot and Bateson 2012).

Pigs kept in the barren housing conditions that are common in intensive pig farming show behavioural and physiological signs of chronic stress (Beattie et al. 2000; Bolhuis et al. 2006; de Jong et al. 2000), which could be accompanied by a negative affective state. Besides, it has been shown that negative experiences in early life can have long-term effects on behaviour, physiology, and cognition (Archard et al. 2012; Bolhuis et al. 2006; Chaby et al. 2013; Lukkes et al. 2009; Munsterhjelm et al. 2009, 2010; Sheriff et al. 2009). The effect of housing on affective state could thus not only depend on the current housing environment, but also on early life housing conditions. For instance, adult rats which were exposed to early life social isolation exhibited increased anxiety and conditioned fear behaviours, later in life (Lukkes et al. 2009). Also in pigs, long-term impacts of early life experiences have been found (see Telkänranta and Edwards 2017 for review), with sometimes favourable effects of enrichment at a young age on later life HPA-axis functioning and a decrease in aggressive behaviour in later life (Munsterhjelm et al. 2009, 2010). However, there are indications that pigs that switch from early life enriched housing to barren housing later on show as much or even more signs of poor welfare as pigs kept in a barren environment from birth onwards (Bolhuis et al. 2004; Munsterhjelm et al. 2009), suggesting that a loss of enrichment may be more detrimental than barren housing throughout life. Thus, apart from a potential long-term negative effect of adverse early life conditions, also a change from a favourable to a suboptimal (barren) environment may result in a negative mood (Burman et al. 2008; Douglas et al. 2012).

Several studies suggest that personality (traits) may interact with affective state (Barnard et al. 2018; Lecorps et al. 2018) and, for instance, affect the attention bias for negative and positive stimuli (Segerstrom 2001). Part of the personality of humans and other animals is revealed in the way which they cope with challenging situations, i.e., their coping style. Coping can be defined as the behavioural and physiological efforts to master a situation (Koolhaas et al. 1999), where successful coping mainly depends on the controllability and predictability of the stressful condition (Koolhaas et al. 1999; Ursin and Olff 1995). Individuals show a wide variation in adaptive coping responses when exposed to the same stressful situation (Bolhuis et al. 2004; Koolhaas et al. 1999). Part of this variation is consistent across time and context, suggesting that the coping style of an individual is a stable personality trait (Koolhaas et al. 1999). Response patterns of individuals at either extreme end of their population are referred to as proactive vs. passive or reactive coping styles (Koolhaas 2008; Koolhaas et al. 1999). Proactive copers tend to use prior experience rather than present information, and are more likely to develop habits, ignore small environmental changes, and, therefore, seem less flexible to adapt to changing situations than reactive copers (Bolhuis et al. 2004; Koolhaas et al. 1999). On the other end of the scale, passive or reactive copers seem to keep a close eye to their environment, respond to even small changes, and more flexibly adjust to changing conditions (Bolhuis et al. 2004; Koolhaas et al. 1999), and may, therefore, more easily adapt to the environment (Coppens et al. 2010). In pigs, a divergence in coping responses can be assessed by exposing them to a back test at an early age. The reaction of piglets to this manual restraint in supine position seems to reveal part of their coping style. The reaction to this test is heritable (Iversen et al. 2017; Velie et al. 2009; Zebunke et al. 2015). Pigs that struggle and squeal relatively much in this test, the “high resisters” (HR) or proactive coping pigs, respond more actively to challenges (Reimert et al. 2013b, 2014b; Ruis et al. 2001), are more rapid (Zebunke et al. 2015) but superficial in their exploration of novel stimuli (Jansen et al. 2009) and poorly adapt to a change in environment (Geverink et al. 2004). Pigs responding with relative immobility and silence in the back test, the “low resisters” (LR) or reactive coping pigs, on the other hand, are more cautious in exploration (Bolhuis et al. 2004; Jansen et al. 2009) and more flexible in adjusting their behaviour to changing conditions (Bolhuis et al. 2004; Geverink et al. 2004; Melotti et al. 2011). It has been suggested that pigs with a reactive coping style are more influenced by their housing environment, i.e., enriched vs. barren (Asher et al. 2016; Bolhuis et al. 2003, 2005a, b). For example, Asher et al. (2016) described that pigs with a more reactive personality responded more pessimistically in a judgement bias test when housed barren, but more optimistically when kept in a more enriched environment. Thus, when assessing the effects of early life and current housing conditions on the affective state of pigs, part of the individual variation found may be related to their personality type or coping style.

The aim of this study was to investigate the impacts of early life and current enrichment on the affective state of pigs using an attention bias test, and their potential interaction with the pigs’ individual coping style.

Our attention bias test for pigs was based on a test recently developed for sheep (Lee et al. 2016; Monk et al. 2018b), involving a food reward as a positive stimulus and the attention paid to a threat as a measure of a negative affective state. This test in sheep has been shown to reflect anxiety as sheep treated with anxiolytic or anxiogenic drugs showed reduced and increased attention to the threat and vigilance, respectively (Lee et al. 2016). In our study, pigs housed in either barren or enriched housing in early life, either experiencing a switch in housing conditions at a later age or not, were exposed to a similar attention bias test. We hypothesized that barren-housed pigs, and particularly those that had experienced enrichment in early life, would pay more attention to the threat, show more vigilance, and would be less willing to eat the food reward, because they were expected to have a more negative affective state (more anxious). Moreover, we predicted that the affective state of LR (reactive) pigs would be more affected by their housing conditions, with barren-housed LR pigs and LR pigs experiencing a loss in enrichment in later life responding most negatively to the threat.

## Materials and methods

The established principles of laboratory animal care and use were followed, as well as the Dutch law on animal experiments. The Animal Care and Use Committee of Wageningen University and Research approved the experiment (DEC code: 2017.W-0001.001.IvD.3).

### Animal and housing

Piglets (*Tempo *× *Topigs 20*) from 30 sows, divided over 2 batches equally and balanced for groups, were studied in this experiment. Sows were inseminated on the same day within a batch. From 1 month before farrowing, they were housed at the experimental facilities (Carus) of Wageningen University and Research, Wageningen, The Netherlands. During the lactation period, 14 litters of piglets and their sows were housed in 8.6 m^2^ barren (B1) pens with a solid floor and a small area with slats. The other 16 litters of piglets were housed in enriched pens (E1) with the same 8.6 m^2^ part as in the barren pens, to which another 8.5 m^2^ area was added (total pen size 17.1 m^2^) that was enriched with 1.7 kg straw, 300 L of sawdust, and 270 L of peat. Extra, fresh straw and sawdust were added daily, and peat was added weekly (0.8 kg/day straw, 40 L/day sawdust, and 30 L/week peat) in the enriched part. In addition, two toys hanging against the wall of the pen, one chain with a ball and one chain with screws that touched floor, were placed in the barren pens, and two toys, one chain with a ball and a toy that was alternated daily selected from four different toys, were placed in the enriched pens from 5 days after birth. All sows were housed in the same farrowing crates without access to the enrichment. In the first week after birth, one heating lamp was provided in the barren pens, and two lamps in the enriched pens. Each pen had one drinking nipple for the piglets and one for the sow. Sows were fed a standard commercial diet twice a day. From 5 days of age onwards, piglets received some fresh commercial feed. Temperature was set at 25 °C and gradually decreased to 21 °C over a course of 2 weeks. Each pen was cleaned daily, and lights and a radio were on from 7:00 until 19:00 h.

At 13 days of age, all piglets were subjected to a back test to assess their coping style, also referred to as personality (Bolhuis et al. 2000). Briefly, in this test, piglets are restrained in supine position for 1 min and the number and latency of escape attempts and vocalisations are recorded (see Melotti et al. 2011 for details). In line with Reimert et al. (2014b), pigs were classified as relatively “high resisters” (HR) if they struggled 2 times and vocalized at least 25 times, or struggled at least 3 times, and as “low resisters” (LR) if they struggled 0 or 1 time, or struggled 2 times and vocalized less than 25 times. In this selection criterion used, no extremes were selected, but, rather, the experimental pigs were either labelled as HR or LR (Melotti et al. 2011).

Pigs were weaned at 28 days of age, and 192 pigs (96 per batch) were selected and regrouped in 32 new pens containing 6 pigs each from different farrowing pens. The composition of each new group was balanced for sex, coping style and bodyweight. Housing treatment (B1 vs. E1) for each pig was kept the same as before weaning; thus, B1 pigs went to barren pens, which measured 5.6 m^2^ and had partly solid floor and partly slatted floor. The other half of pigs from E1 farrowing pens were housed in 11.2 m^2^ enriched pens with 2.5 kg straw, 400 L of sawdust, and 360 L of peat on the floor. Extra, fresh straw and sawdust were added daily, and peat was added weekly in enriched pens (1.25 kg/day straw, 60 L/day sawdust, and 45 L/week peat). From 39 days of age, enriched housed pigs also received extra enrichment (e.g., branches, jute sacks, egg trays, and ropes) on each Monday until the end of the experiment.

Each pen had one drinking nipple and pigs received solid commercial feed ad libitum. On the weaning day, the temperature was set at 25 °C and was decreased over the course of 2 weeks to 21 °C. It was kept at 21 °C until the end of the experiment. After weaning, one heating lamp was provided in both barren and enriched pens for 2 weeks. Lights and a radio (channel with Dutch pop music and hourly news) were on from 7:00 until 19:00 h.

At 47 days of age, pigs experienced either a switch in housing conditions (from barren to enriched or vice versa) or not, resulting in four treatment groups, E1E2, E1B2, B1E2, B1B2, *n* = 8 pens, and 48 pigs per group. B1B2 and E1E2 groups were also moved to new pens to control for handling and relocation effects, but without a change in enrichment conditions. After this switch, straw, peat, and toys were provided and added as described before, but only 30 L of sawdust was added daily.

### Attention bias test

To assess attention bias in pigs, which could be an indicator of their affective state (negative valence related to anxiety), an attention bias test was carried out with 128 pigs, when they were around 76 days of age. Four pigs per pen were selected from E1E2, E1B2, B1E2, and B1B2 housing conditions, with equal numbers of each sex and coping style.

Half of the pigs were exposed to a (non-social, unfamiliar) threat as experimental group, and the other half were not and served as control group, balanced for sex and housing conditions.

**Fig. 1:**
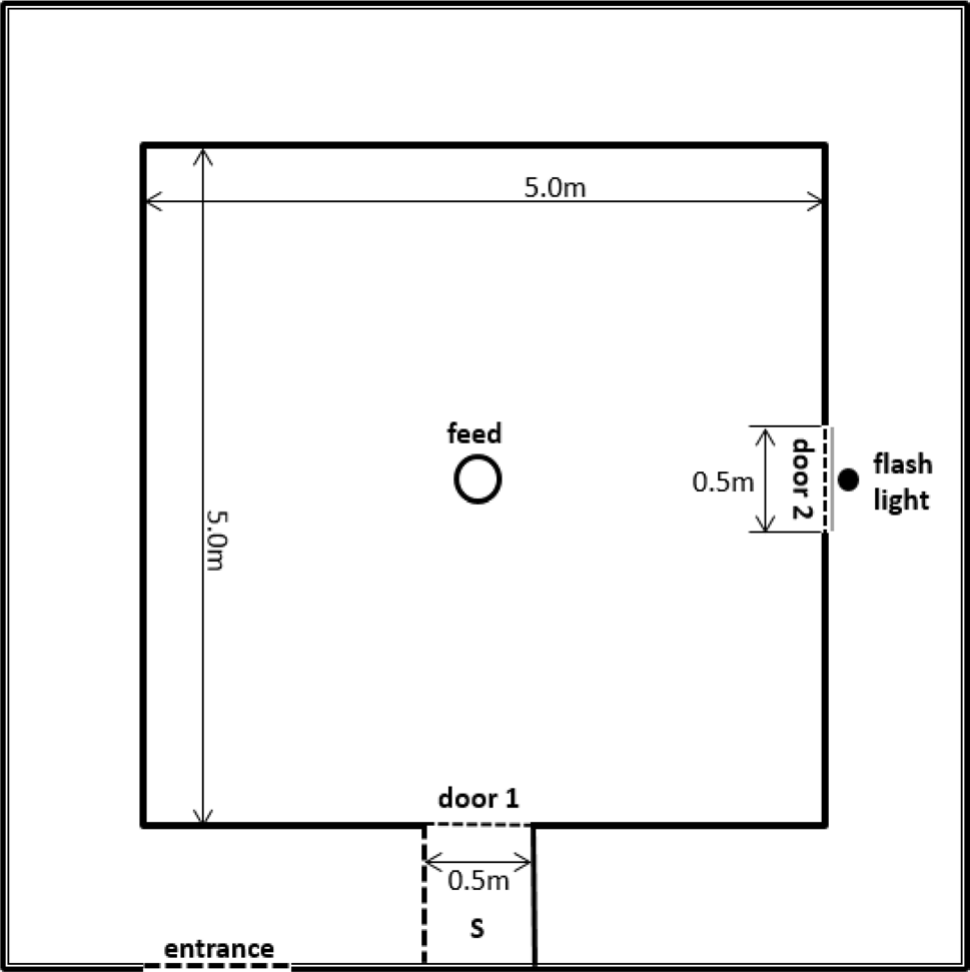
Layout of the room used for the attention bias test. S indicates a start box. Door 1 is the entrance and exit door to the test arena, and door 2 is a guillotine door in front of the plexiglass behind which the flash light was placed

The test set-up was based on the sheep study from Lee et al. (2016). Pigs were individually placed in a 5 × 5 m test arena with solid walls away from their home pen (Fig. 1). All pigs had been in the arena once before for another test at 6 weeks of age. In the centre of the arena, a metal bucket was placed containing feed (450 g of the pigs’ normal feed) to which eight chocolate peanuts and ten pieces of carrots were added (which are generally preferred over normal feed). Testing order was balanced for housing, sex, and coping style.

At the beginning of the test, a red and blue flash light (off) was present outside the arena and was visible through a plexiglass window (0.5 m^2^), when guillotine door 2 was lifted, on the right side of the arena. Once a pig entered the test arena from door 1, the door was shut behind it, and a timer was started. When the pig looked at the direction of the threat or maximum 10 s after the pig entered the test arena, the flash light was turned on and door 2 was pulled up and down continuously (seven times), producing loud squeaking sounds, rapid movement while showing the flash light. This threat, i.e., the combination of the flash light with the moving door and aversive sound, lasted for 10 s. Hereafter, door 2 was softly closed and the flash light was turned off. Pigs stayed in the test arena for 180 s in total and left the test arena from door 1 to go back to their home pen. The test was carried out over 2 days per batch.

Behaviour of the pigs, as well as attention towards the threat, were scored continuously using behaviour sampling with The Observer 5.0 software (Noldus Information Technology b.v., the Netherlands) (see Table 1 for the ethogram). In addition, the latency to eat after the threat had been removed was scored. One observer scored vocalisations, defecating and urinating of all pigs. Another single observer recorded all other behaviours of the pigs. Interrater reliability of behavioural observations was deemed substantial (Cohen’s kappa = 0.74). After removal of the threat, 17 pigs failed to eat and their latency was set at the maximum time score.

**Table 1:** Ethogram used to score the behaviours of the pigs during the attention bias test

Behaviour	Definition
Attention class	
Attention to the threat	With the head oriented toward the location of the threat
Attention to something else	With the head oriented toward other directions, except threat
Behaviour class	
Vigilance	Standing motionless with head at shoulder height or higher or lower
Eating	Eating food in the bucket. The eating event continues, while the pig is chewing provided that the head stays close to the bucket and the pig remains non-vigilant. Once the pig becomes vigilant or moves away from the bucket, this is considered to be the end of eating, even if the pig continues chewing
Exploring	Exploring the floor or wall of the arena by sniffing, nosing, licking or rooting it with the rooting disc
Walking	Walking without performing any other described behaviour. All four legs move or the pig turns around at the same spot without moving all four legs
Standing	Standing with four paws on the floor without performing any other described behaviour
High-pitched vocalizations^a^	Screams, squeals, or grunt-squeals
Short grunt^a^	Grunt lasting less than 0.4 s (Fraser 1974; Kiley 1972)
Long grunt^a^	Grunt lasting more than 0.4 s (Fraser 1974; Kiley 1972)
Bark^a^	A low tone that sounds like “wuff”
Defecating^a^	Defecating
Urinating^a^	Urinating
Escaping^a^	The pig jumps to the wall or the door

Behaviours were scored as states unless indicated otherwise

^a^Were scored as events, all other behaviours as states

### Statistical analyses

SAS (SAS 9.4, SAS Institute Inc.) was used for all statistical analyses. Preliminary analyses revealed no effects of testing day, and therefore, it was removed from the final models. Urinating was very rare (*n* = 12 pigs, after threat), and escape behaviour and barks were not observed at all and were, therefore, not analysed. Preliminary analysis also revealed that short grunts were rather rare (only 16% of all grunts in pigs exposed to the threat). Therefore, short and long grunts were summed as ‘grunts’.

For assessing the effect of the threat, we first compared the behaviours of the threat vs. the non-threat (control) pigs over the whole 180 s test. To that end, a mixed linear model with presence of the threat and batch as fixed effects, and pen nested within pre-housing (i.e., housing before the switch at 47 days of age), post-housing (i.e., housing after the switch which was the current housing at the time of the attention bias test), and batch as random effect was used. High-pitched vocalisations were analysed as a 0–1 variable using a generalized mixed model with a binary distribution and logit link function.

Subsequently, we analysed the behaviours of the threat pigs both during the 10-s threat and for a 150-s period after the threat. For this, a mixed linear model was used with pre-housing, post-housing, coping style, their interactions, and batch as fixed effects, and pen nested within pre-housing, post-housing, and batch as random effect. Rates of grunts per min and frequency of vigilance after the threat were square root transformed to obtain normality of residuals. In one of the treatment combination, no high-pitched vocalisations occurred at all. Therefore, high-pitched vocalisations were analysed as a 0–1 variable using Fisher’s exact tests to compare treatment groups. Sex did not affect any of the variables and was, therefore, removed from the final models.

Significant interactions (*p *< 0.05) were further investigated with post hoc pairwise comparisons using the least square means, with Tukey correction for three-way interactions. Results are presented as mean ± SEM.

## Results

### Comparison of threat vs. non-threat (control) pigs

Table 2 presents the behaviours of pigs exposed and pigs not exposed to the threat. Pigs that were exposed to the threat paid attention to the location of the threat for a longer time (*F*_1,95_ = 144.7*, p* < 0.001). Besides, pigs that were exposed to the threat spent more time on vigilance behaviour (*F*_1,95_ = 19.8, *p* < 0.001), and walking (*F*_1,95_ = 6.5, *p* = 0.013), less time on eating (*F*_1,95_ = 19.0, *p* < 0.001), and were more likely to utter high-pitched vocalisations (*F*_1,95_ = 7.9, *p* = 0.006) than pigs without a threat during the 180 s test. No effects were found on time spent exploring, standing, and on rates of grunting or defecating.

**Table 2:** Means ± SEM of the behaviours and vocalisations of pigs that were exposed to a threat and pigs that did not experience a threat during the 3-min test

Behaviour	Threat	No threat	*p* value
Attention to the threat (% of time)	7.1 ± 0.6	0.5 ± 0.1	< 0.001***
Vigilance (% of time)	13.6 ± 1.4	6.7 ± 1.0	< 0.001***
Eating (% of time)	15.0 ± 1.8	27.8 ± 2.4	< 0.001***
Exploring (% of time)	39.4 ± 2.6	37.7 ± 2.5	0.624
Walking (% of time)	19.6 ± 1.6	14.6 ± 1.2	0.013*
Standing (% of time)	12.2 ± 1.3	13.0 ± 1.3	0.677
High-pitched vocalisations (% of pigs)	29.7	9.4	0.006**
Total grunts (rpm)	5.9 ± 0.9	4.5 ± 0.6	0.705
Defecation (rpm)	0.49 ± 0.06	0.42 ± 0.05	0.375

***p < 0.001, **p < 0.01, *p < 0.05

### Behaviour of the pigs exposed to the threat

**Fig. 2:**
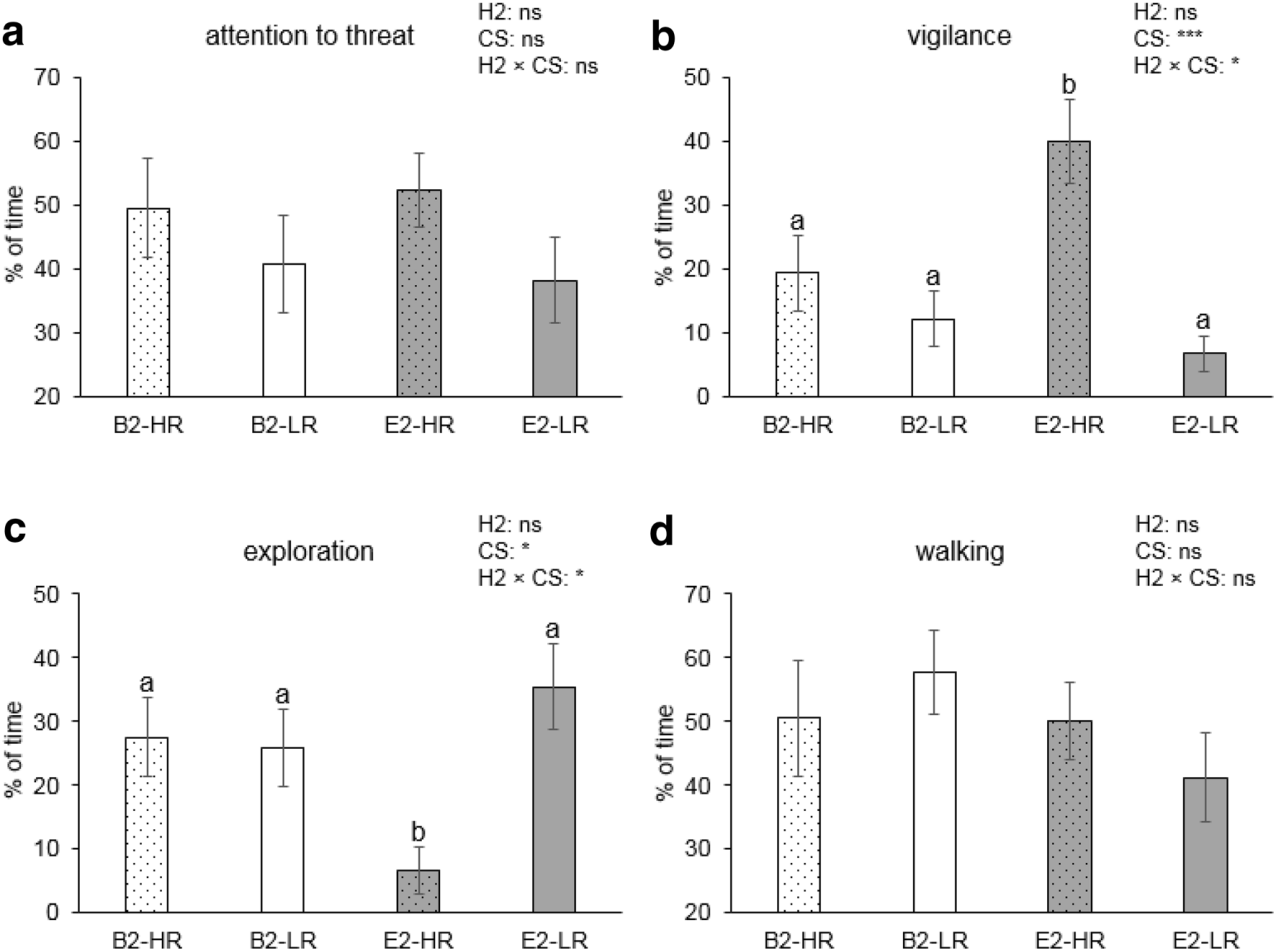
Percentages (mean ± SEM) of time spent on behaviours during the presence of the threat for high-resister (HR) vs. low-resister (LR) pigs housed in barren (B2) and enriched (E2) conditions. B2 and E2 refer to all pigs in barren housing or enriched housing from 7 weeks of age, respectively, irrespective of their previous housing. **a**–**d** The percentage of time spent on attention towards the threat, vigilance, exploration, and walking, respectively. Significances of post-housing (H2), coping style (CS), and the post-housing × coping style (H2 × CS) interaction are indicated by ***p < 0.001, *p < 0.05, and non-significance is ns. Groups lacking a common letter (a, b) significantly differ (*p *< 0.05)

**Fig. 3:**
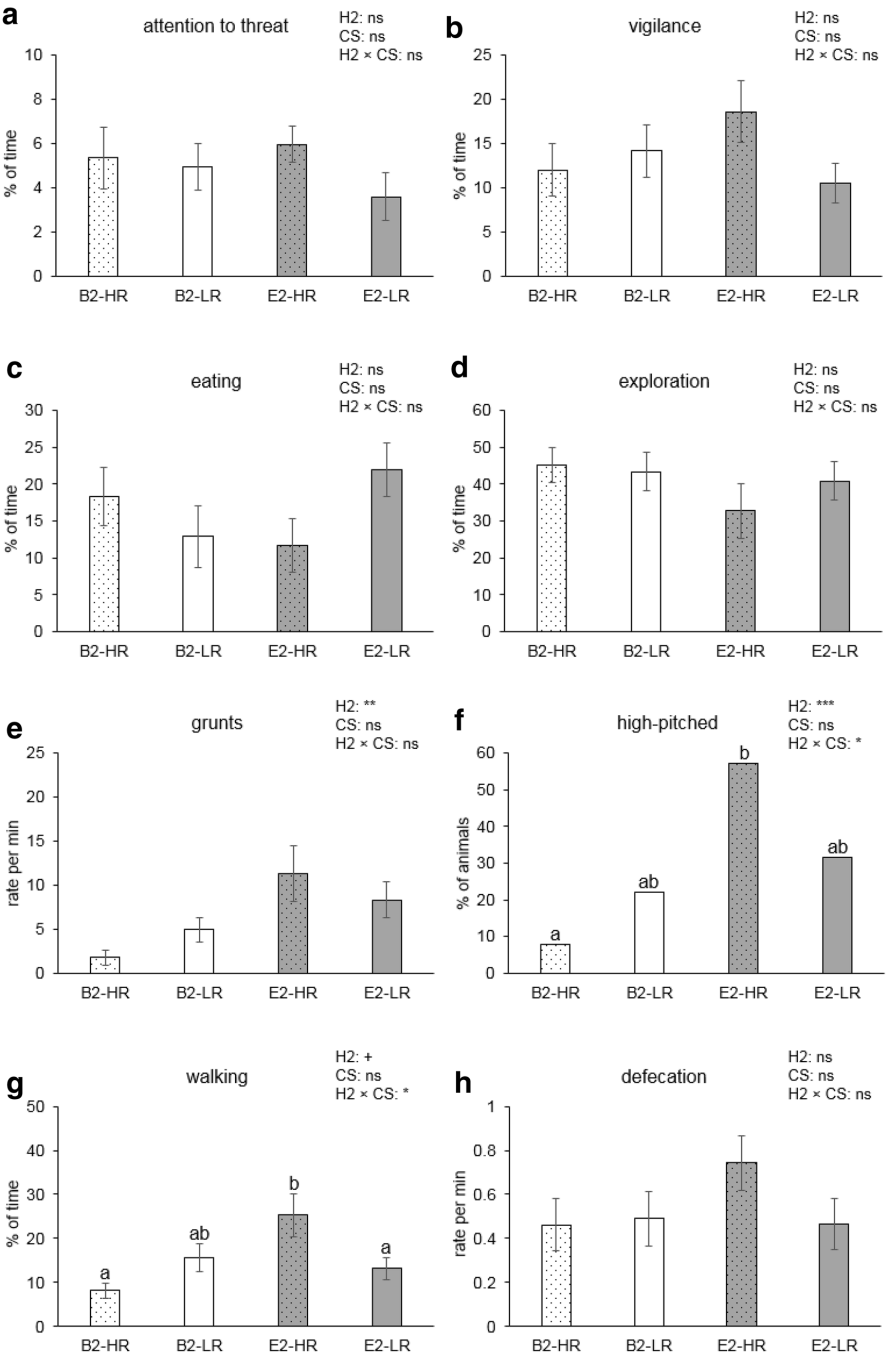
Means ± SEM of behaviours after removal of the threat (150 s) for high-resister (HR) vs. low-resister (LR) pigs housed in barren (B2) and enriched (E2) conditions. B2 and E2 refer to all pigs in barren housing or enriched housing from 7 weeks of age, respectively, irrespective of their previous housing. **a**–**d**, **g** The percentage of time spent on attention towards the threat, vigilance, eating, exploration, and walking, respectively; **e**, **h** the rate of grunts and defecation per min, and **f** shows the percentage of pigs making high-pitched vocalisations. Significances of post-housing (H2), coping style (CS), and the post-housing × coping style (H2 × CS) interaction are indicated by ***p < 0.001, **p < 0.01, and *p < 0.05; tendency is indicated by ^+^*p *< 0.10, and non-significance is ns. Groups lacking a common letter (a, b) significantly differ (*p *< 0.05)

**Fig. 4:**
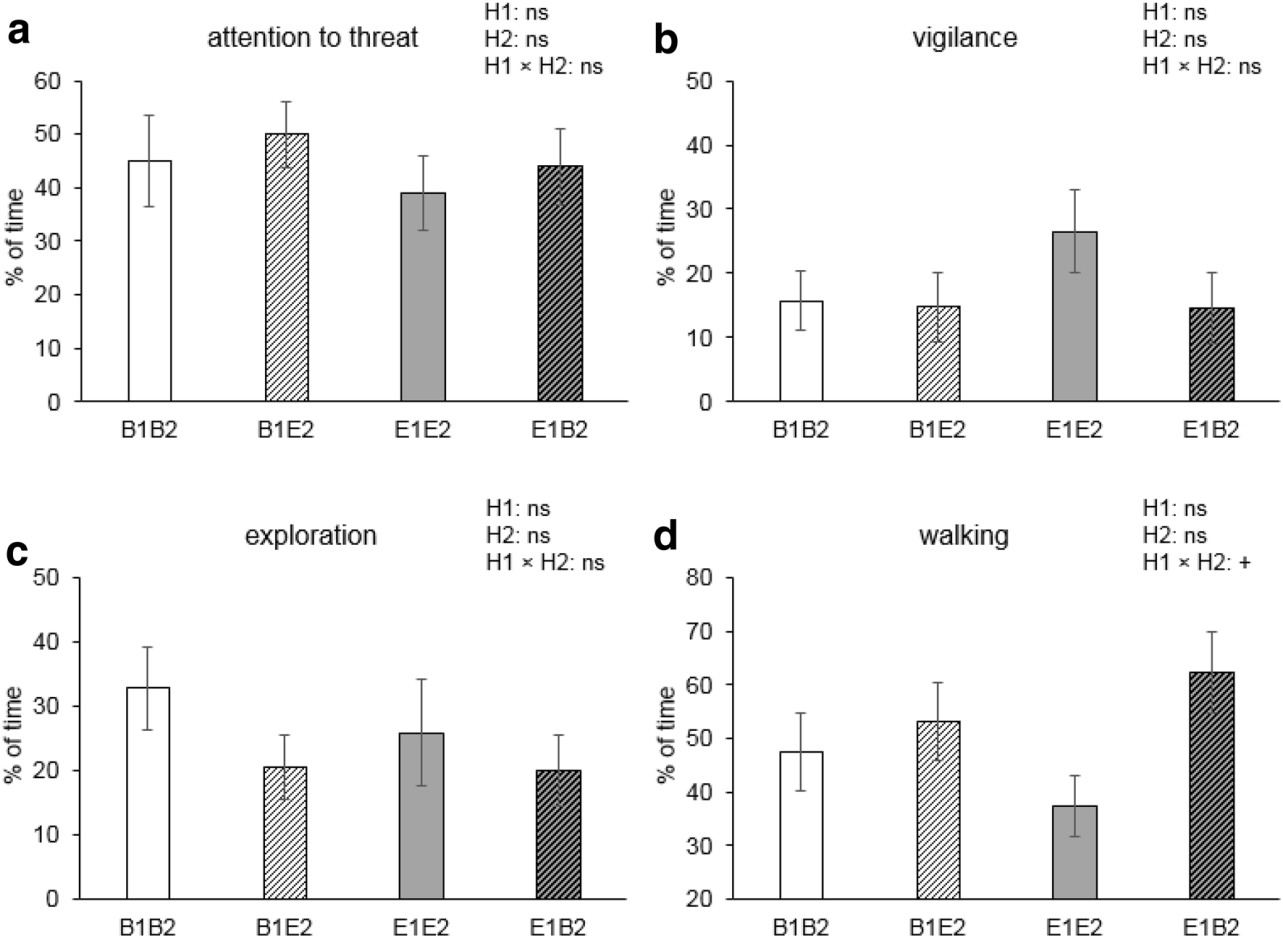
Percentages (mean ± SEM) of time spent on behaviours during the presence of the threat for pigs housed in four different housing conditions (B1B2 and E1E2: housed in barren and enriched pens, respectively, throughout life; B1E2 and E1B2: experienced a switch in housing conditions from barren to enriched or vice versa at 7 weeks of age). **a**–**d** The percentage of time spent on attention towards the threat, vigilance, exploration, and walking for pigs during the presence of the threat. Tendency of the pre-housing × post-housing (H1 × H2) interaction is indicated by ^+^*p *< 0.10, and non-significance is ns

**Fig. 5:**
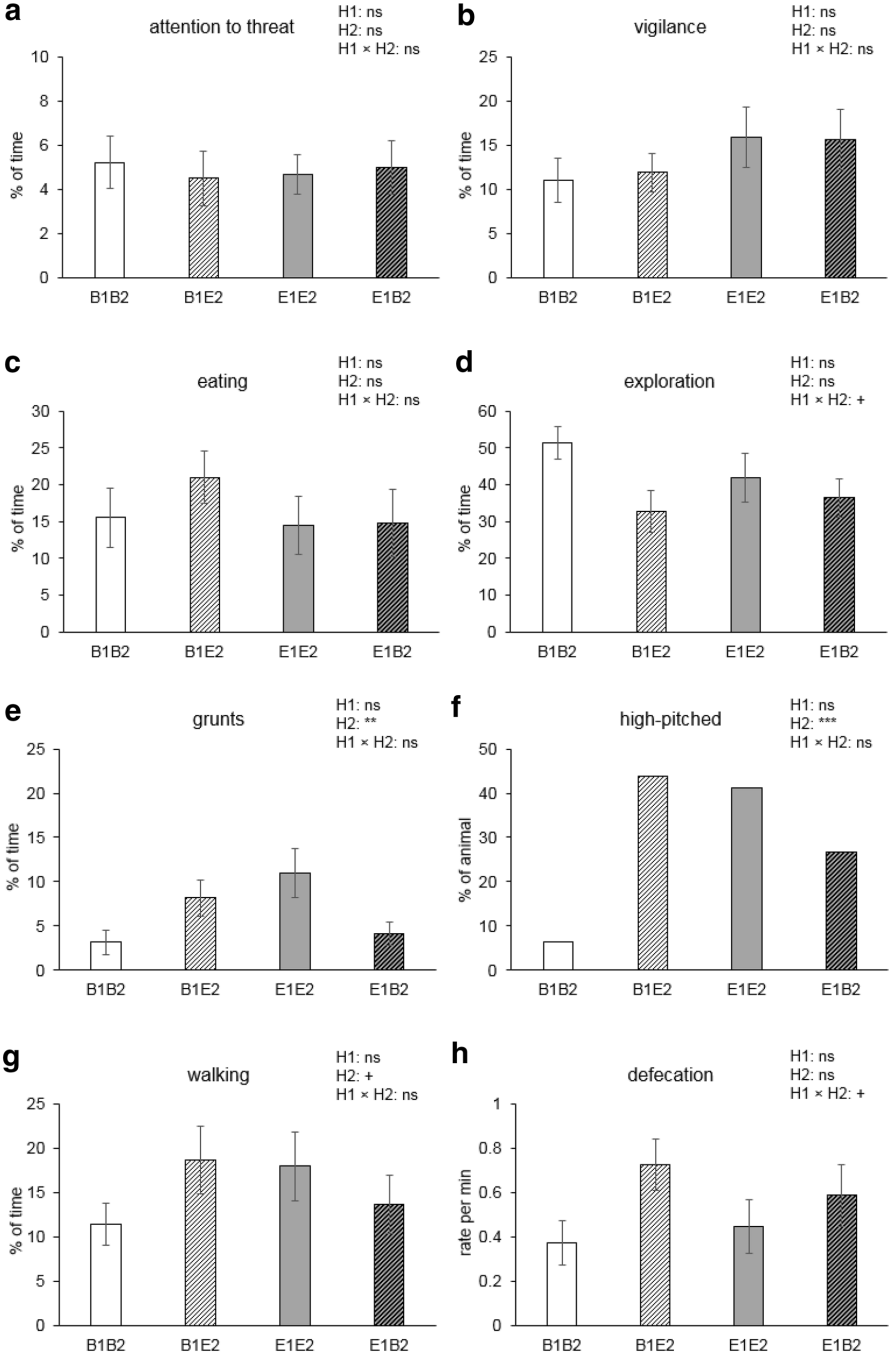
Means ± SEM of behaviours for pigs housed in four different housing conditions (B1B2 and E1E2: housed in barren and enriched pens, respectively, throughout life; B1E2 and E1B2: experienced a switch in housing conditions from barren to enriched or vice versa from 7 weeks of age). **a**–**d**, **g** The percentage of time spent on attention towards the threat, vigilance, eating, exploration, and walking, respectively; **e**, **h** the rate of grunts and defecation per min; and **f** shows the percentage of pigs making high-pitched vocalisations. Significances of pre-housing (H1), post-housing (H2), and the pre-housing × post-housing (H1 × H2) interaction are indicated by ***p < 0.001, **p < 0.01; tendency is indicated by ^+^*p *< 0.10, and non-significance is ns

Behaviour of pigs exposed to the threat was mainly influenced by (trends for) the interaction between post-housing and coping style, and the interaction between pre-housing and post-housing, whereas only one three-way interaction was found (as described in the text below). Therefore, the post-housing × coping style interaction is highlighted in Figs. 2 and 3; the pre-housing × post-housing interaction is shown in Figs. 4 and 5.

### Behaviour of the pigs during the 10-s threat

Only 6 out of 64 pigs ate during the threat and only 9 out of 64 pigs showed the behaviour standing. Pigs did not vocalize or defecate during the threat.

Attention towards the threat tended to be affected by the pre-housing × coping style interaction (*F*_1,27_ = 3.1, *p* = 0.092). Time spent on vigilance behaviour was affected by coping style (*F*_1,27_ = 15.6, *p* < 0.001) and the post-housing × coping style interaction (*F*_1,27_ = 5.8, *p* = 0.023). Post hoc analysis showed that E2-HR pigs showed more vigilance behaviour than other pigs (*p* < 0.05, Fig. 2b). Exploration was affected by coping style (*F*_1,27_ = 5.2, *p* = 0.031) and the post-housing × coping style interaction (*F*_1,27_ = 6.7, *p* = 0.014) and the pre-housing × post-housing × coping style interaction (*F*_1,27_ = 4.8, *p* = 0.036). E2-HR pigs spent less time on exploring than other pigs (*p* < 0.05, Fig. 2c), but this effect was merely due to the large coping style contrast within E1E2 housing: HR pigs in E1E2 housing (1.9 ± 0.9%) explored much less than their E1E2-LR counterparts (47.2 ± 11.8%, *p *= 0.012). The percentage of time spent on walking tended to be affected by the pre-housing × post-housing interaction (*F*_1,27_ = 4.2, *p *= 0.050).

### After the threat

There were no housing or coping style effects on time spent on attention to the threat location, vigilance, and eating after the threat was removed. The frequency of paying attention to the threat, however, was affected by the post-housing × coping style interaction (*F*_1,28_ = 5.1, *p* = 0.031), with higher levels for E2-HR (2.6 ± 0.4) than for E2-LR pigs (1.4 ± 0.3, *p *= 0.045) and levels of B2 pigs in between (B2-HR 1.7 ± 0.4; B2-LR 2.3 ± 0.5). Changing from vigilance to other behaviours tended to be affected by post-housing × coping style (*F*_1,28_ = 3.2, *p *= 0.086). E2 pigs tended to start eating sooner (63.4 ± 9.2 s) than B2 pigs after the threat had ended (95.3 ± 10.1 s, *F*_1,27_ = 4.1, *p *= 0.052) and exploring tended to be affected by the pre-housing × post-housing interaction (*F*_1,27_ = 3.6, *p *= 0.068). Walking tended to be affected by post-housing (*F*_1,27_ = 4.0, *p *= 0.057), and was affected by the post-housing × coping style interaction (*F*_1,27_ = 6.3, *p *= 0.018). Post hoc analysis showed that E2-HR pigs walked more than E2-LR pigs (*p *= 0.029) and B2-HR pigs (*p *= 0.006, Fig. 3g). The percentage of time spent on standing was affected by pre-housing × post-housing (*F*_1,27_ = 4.8, *p *= 0.038). E1B2 pigs (19.4 ± 4.1%) stood more than E1E2 pigs (9.7 ± 1.6%, *p *= 0.047), with levels of B1B2 pigs (10.4 ± 2.6%) and B1E2 pigs (15.6 ± 2.7%) in between. E2 pigs grunted more often (9.8 ± 2.0 per min) than B2 pigs (3.5 ± 0.9 per min) (*F*_1,27_ = 9.1, *p *= 0.006). The proportion of pigs that displayed high-pitched vocalisations was also higher in E2 housing than in B2 housing (*F*_1,27_ = 6.5, *p *= 0.029), but only so in the HR pigs (*p *= 0.013 for post-housing effect within HR pigs, see Fig. 3f). The rate of defecating per min tended to be affected by the pre-housing × post-housing interaction (*F*_1,27_ = 3.7, *p *= 0.065).

## Discussion

In this study, we investigated the impacts of early life and current enrichment on attention bias in pigs with diverging coping styles. We hypothesized that pigs from barren housing conditions, especially those that had experienced enrichment in early life, would pay more attention to the non-social, unfamiliar threatening stimulus, show more vigilance, and would be less willing to eat, because they were expected to have a more negative, anxious affective state. Besides, LR (reactive) pigs were expected to be more affected by their housing environment than HR (proactive) pigs.

### Response to the threat

Pigs exposed to the 10-s threat approximately doubled the time spent on vigilance behaviour and halved time spent eating. Moreover, they paid more attention towards the location where the threat had been in the test room than pigs that were not exposed to the threat, and were more likely to display high-pitched vocalisations, which indicates that the pigs clearly responded to the threat, also after it had disappeared. It has been shown in the previous studies that animals with a more negative affective state were more vigilant, less willing to eat, and paid more attention to the location of a threat (Brilot and Bateson 2012; Lee et al. 2016, 2018), which is parallel with what we found in this study, and confirms that the threat, a combination of a flash light and a moving guillotine door producing squeaking sounds, was aversive for pigs. Similar to human studies (Bar-Haim et al. 2007) and sheep studies (Lee et al. 2016; Monk et al. 2018b), attention towards a threat and vigilance were our key measures of attentional-orienting.

### Effects of early life conditions

Based on the previous behavioural studies (Bolhuis et al. 2005a; Douglas et al. 2012), we expected barren-housed pigs (B2) to be in a more negative affective state than enriched housed pigs and thus to pay more attention to the threat (measured as head orientation) and be more vigilant (see below for discussion on effects of current housing). We furthermore hypothesized this to be even more so for pigs originating from an enriched early life environment (E1B2) that thus had experienced a downgrade change in housing conditions (Bolhuis et al. 2005a; Douglas et al. 2012; Munsterhjelm et al. 2009). The absence of interactions between early and later life housing, and the absence of an effect on attention to the threat or general vigilance, however, do not point to such an effect of a negative switch. Besides, we found no indications of a beneficial effect of early life enrichment either, which could mean that housing conditions in early life do not have long-term effects on affective state, or at least not on anxiety. Alternatively, potential effects of early life housing (up till 7 weeks of age) on the behavioural responses in the test may have been overruled by the later housing conditions which the pigs had been kept in for 4 weeks at the time of attention bias testing (11 weeks of age). In another study, we did find, though, that pigs from enriched housing in early life responded less strongly to reward loss in a successive negative contrast test carried out 8 weeks after a change in housing conditions (Luo et al., submitted), which could point to a more positive affective state in these animals.

### Effects of current housing

The effect of later life environmental conditions, i.e., housing at the time of testing, on behaviour in the attention bias test was also not in line with our expectations and other studies, and, moreover, partly depended on coping style. During the threat, enriched housed HR pigs were more vigilant than other pigs. They also spent less time on exploration, particularly the HR pigs kept in enriched pens from birth onwards. After the threat, no effects were found on time spent on attention to the (location of the) threat or vigilance, but HR pigs from enriched pens more frequently paid attention to the threat than LR pigs from enriched pens. Enriched pigs grunted more often and, within the HR pigs, they also were more likely to display high-pitched vocalisations like squeals. Increased grunting and squealing have been described to occur during aversive events in which pigs were deprived of (visual and tactile) social contact (Reimert et al. 2013a). Especially, high-frequency calls seem to be indicative of fear and other negative emotions in pigs when socially isolated (Leliveld et al. 2017). Long grunts (also called low grunts (Leliveld et al. 2017) were the most uttered vocalisations during the test and have been suggested to be used in social isolation as an attempt to make contact with group members (Kiley 1972; Leliveld et al. 2017). Possibly, the higher grunting rate in E2 pigs is related to a higher motivation to get into contact with their pen mates. Taken together, with the exception of a trend for a longer latency to start eating after the threat in barren-housed pigs as compared with pigs from enriched housing, our results do not point to a more negative long-term affective (anxious) state in enriched animals. This could mean that barren housing does not induce a long-term negative mood. This seems unlikely, though, given the results of previous studies. Barren-housed pigs have been reported to experience more chronic stress (Bolhuis et al. 2005a, 2006), and to have a more negative affective state (Douglas et al. 2012) than enriched housed pigs, although it should be noted that another study reported no effects of barren housing on judgement bias in spite of clear negative effects on physiological and behavioural welfare indicators (Carreras et al. 2016). In a study in sheep, chronic stress, which was confirmed by HPA-axis dysregulation, against expectations reduced rather than increased vigilance to a predator threat (Verbeek et al. 2019). Explanations given for the lack of effects or effects opposite to expectations in this sheep study and in a quail study were a poor sensitivity of the test, or the context of the test (e.g., test arena and handling) which may have overruled the putative long-term effects of housing conditions on mood (Horváth et al. 2016; Verbeek et al. 2019). The latter could hold for our study as well, as pigs from barren housing may have experienced the release from their suboptimal environment to the test arena as more positive than enriched housed pigs. Several studies suggest that negative events, such as sheering (Sanger et al. 2011) and restraint plus social isolation in sheep (Doyle et al. 2010), before testing, result in more positive affective states during testing (i.e., a positive contrast between the test situation and the previous situation). It is possible that a long-term negative situation (like barren housing) preceding testing has a similar effect. In support of this, a study on rats reported a relatively positive affective state of barren-housed individuals during a mood test (Mitchell et al. 2012).

When investigating the impact of housing conditions in group-housed animals on attention bias, the behavioural response in the test might, thus, potentially be influenced by the relative contrast between the test room/situation and the home environment, as well as by social isolation. Indeed, in the most convincing studies on the effect of mood on attention bias towards threatening stimuli, negative affective states were induced by pharmacological interventions [e.g., by administration of diazepam and m-CPP) in sheep (Lee et al. 2016; Monk et al. 2018b) and rats (Wright and Rodgers 2014)]. In these studies, the relative contrast between the home and test environment was the same for all animals, and the drugs remained active for the duration of the test, which likely circumvented a potential short-term influence of the test context on emotional state. Furthermore, Verbeek et al. (2019) suggested that chronic stress may alter the motivation to obtain feed rewards which could also interfere with the test outcome. In a successive negative contrast test, we did find indications for a lower reward sensitivity in pigs housed in barren conditions from birth onwards as compared with pigs that had switched from enriched to barren pens and with enriched housed pigs (Luo et al., submitted). Finally, it has been suggested that individuals suffering from chronic stress or depression are less responsive to stimuli—either negative or positive—in general (Fureix et al. 2012) which could also explain the lack of a clear housing effect on attention bias.

### Effects of coping style and its interaction with housing

In humans, cognitive biases are dependent on both current mood and personality (Marshall et al. 1992). Classically, humans scoring high on the personality dimension neuroticism more often and strongly experience negative affect, whereas extraversion is associated with frequent and intense expression of positive emotions (Winter and Kuiper 1997). These personality traits, thus, possibly impact long-term mood. We studied a personality trait in pigs, i.e., their coping style, which is not inherently expected to be linked with a bias towards either negative effect or positive affect. Indeed, it has been suggested that the dimension of coping style is independent of an emotionality dimension (Koolhaas et al. 2007) and rather reflects how individuals respond when challenged. Proactive, i.e., HR, pigs consistently have been shown to vocalize more often in challenging situations (Geverink et al. 2002; Jansen et al. 2009; Reimert et al. 2014a, b; Ruis et al. 2001) than reactive, i.e., LR, pigs, and to display more locomotion (Jansen et al. 2009; Reimert et al. 2013b, 2014b). LR pigs, on the other hand, have been reported to show more vigilance (Reimert et al. 2014a) and continue to do so even in an increasingly familiar environment (Jansen et al. 2009). Both of these response patterns can lead to successful coping with a challenging situation, with the style of reactive copers being more suited for changing environments.

In our study, the impact of housing on the behaviour of the pigs during the test depended on their coping style (and vice versa), in spite of the fact that we did not select the extremes of the tested population but labelled all pigs. We found the highest levels of walking and squealing in HR pigs from enriched housing after removal of the threat. This could, on one hand, reflect the typical mode of responding of HR pigs, or, alternatively, indicate a more negative affective (anxious) state as compared with barren-housed pigs and enriched housed LR pigs. We tentatively suggest the latter, as enriched HR pigs also showed significantly more vigilance during the threat and less exploration. In addition, after the threat, they more often paid attention to the threat than LR pigs from enriched housing, and numerically spent the most time on vigilance and attention towards the location of the threat and had the highest frequency of defecating, which all have been linked with aversive situations and/or seem to reflect negative emotions (Bouissou and Vandenheede 1995; Brilot and Bateson 2012; Lee et al. 2016; Reimert et al. 2013a). The higher impact of housing on the behaviour of HR pigs in this test is not in line with studies, suggesting that proactive copers are less responsive to the distinction between barren and enriched housing than reactive copers. For instance, LR pigs were reported to show more play behaviour in enriched pens, but more oral manipulation of pen mates (e.g., tail and ear biting) and gastric lesions in barren pens than HR pigs (Bolhuis et al. 2005a, 2006). Moreover, Asher et al. (2016) recently found reactive copers to respond more optimistically in a judgement bias test when housed enriched and more pessimistically when kept in barren pens, suggesting that, apart from behaviour, also the affective state of reactive copers is more affected by (lack of) enrichment than that of proactive copers.

It should be noted, though, that HR pigs, albeit seemingly less affected by barren housing, show more difficulty in coping with relocation and social isolation (Bolhuis et al. 2004; Geverink et al. 2004; Ruis et al. 2001). If the behaviour in our test reflected the acute response to the test setting (involving relocation and social isolation) rather than the long-term impact of housing conditions on affective state, this might explain why (enriched) HR pigs were most affected. As argued before, in barren pigs, this effect may have been attenuated by the exposure to a spacious, stimulus-rich test room contrasting their small, barren home pens. Therefore, for future studies on mood in animals, it is worthwhile to consider that (1) personality traits (e.g., anxiety) may influence a long-term affective state per se; (2) personality traits (e.g., coping style) may, irrespective of affective state, influence the mode of responding of animals which could interfere with the read-out variables of a test for affective state; (3) personality traits may influence both the impact of the long-term condition (e.g., housing and chronic stress) under study, as well as that of the test setting.

### Remarks on the test design and measurements

Our test set-up was based on a paradigm developed for sheep that has been successfully validated by pharmacological interventions (Lee et al. 2016; Monk et al. 2018a). In the sheep studies, a dog was effectively used as a threatening stimulus [albeit habituation to dogs in daily life may reduce its threatening potential for sheep, as argued by Verbeek et al. (2019)]. To our knowledge, this is the first study on attention bias in pigs, for which it is harder to find a threatening stimulus. We used a squeaking moving guillotine door and a flash light. We found clear evidence, by comparison with pigs not exposed to this threat, that the pigs were negatively affected by this stimulus, even after its disappearance, but we cannot rule out that stronger and/or longer threats would have given more clear results. In addition, attention towards the threat was in our study based on the position of the head (oriented toward the location of the threat) and not on glancing. In human studies, however, ‘attention’ refers to overt visual attention, typically based on eye gaze (Crump et al. 2018), which we found more difficult to score in an objective manner, but may, nevertheless, have been a more accurate indicator of attention bias. This could be a common concern for previously published studies in freely moving farm animals, in which attention was also measured as the ‘head oriented towards the threat’ (e.g., Lee et al. 2016; Verbeek et al. 2019). Thus, rather than scoring attention bias in the narrow sense (referring to vision), our test may, thus, have picked up general emotional reactivity to the threat, which might alternatively be referred to as ‘altered perception of the threat’. It should be noted, though, that our threat stimulus comprised both visual and auditory cues, and that for pigs (and several other farm animals), other sensory modalities may be as much or even more relevant than vision alone, perhaps, asking for a more broad definition of attention bias and corresponding measurements. Finally, food was used as a positive stimulus, but motivation for food can vary considerably between animals and within animals over the span of a single day (Monk et al. 2018a), and potentially be affected by housing or stress (see above), and so, even though we balanced the test order for our treatments, food may not be an optimal positive stimulus in an attention bias test for pigs. Further studies are required to develop a practical attention bias test in pigs, and to circumvent (or reliably pinpoint) potential confounding effects of the test set-up and test read-outs with personality traits and the long-term treatments, like housing conditions, under study.

## Conclusion

Pigs responded to a short-lived threat during an attention bias test by increasing vigilance, head-orientation towards the (previous) location of the threat, high-pitched vocalisations and reduced time spent eating, revealing increased anxiety. Current, but not early life, housing affected the behaviour in this test in a personality-dependent manner. Although no effects on duration of attention to the threat or vigilance were found, other behavioural variables (e.g., the frequency of attention toward the threat and high-pitched vocalisations) suggest a more negative emotional state following exposure to the threat in HR pigs from enriched housing. The housing effect was opposite to what we expected, which might be explained by a short-term effect of the relative contrast between the home pens of the pigs and the test room. This potentially overruled putative long-term effects of environmental conditions on mood.
